# High Level Serum Procalcitonin Associated Gouty Arthritis Susceptibility: From a Southern Chinese Han Population

**DOI:** 10.1371/journal.pone.0132855

**Published:** 2015-07-16

**Authors:** Wen Liu, Keshav Raj Sigdel, Ying Wang, Qun Su, Yan Huang, Yan Lin Zhang, Jie Chen, Lihua Duan, Guixiu Shi

**Affiliations:** 1 Department of Rheumatology and Clinical Immunology, The First Affiliated Hospital of Xiamen University, Xiamen, Fujian, China; 2 Department of Nephrology, The First Affiliated Hospital of Xiamen University, Xiamen, Fujian, China; 3 Department of Immunology, College of Medicine, Xiamen University, Fujian, China; Penn State University, UNITED STATES

## Abstract

**Objectives:**

To study the serum Procalcitonin (PCT) level in inflammatory arthritis including gouty arthritis (GA), Rheumatoid arthritis (RA), and ankylosing spondylitis (AS) without any evidence of infection were evaluated the possible discriminative role of PCT in gouty arthritis susceptibility in southern Chinese Han Population.

**Material and Methods:**

From Feb, 2012 to Feb, 2015, 51 patients with GA, 37 patients with RA, 41 patients with AS and 33 healthy control were enrolled in this study with no evidence of infections. The serum level of PCT (normal range < 0.05 ng/ml) was measured by electrochemiluminescence immunoassay (ECLIA). Disease activity was determined by scores of VAS (4.07 ± 1.15), DAS28 (4.97 ± 1.12), and ASDAS (2.97 ± 0.81) in GA, RA and AS groups respectively. Other laboratory parameters such as, serum creatinine (CRE), erythrocyte sedimentation rate (ESR), C-reactive protein (CRP), uric acid (UA) and white blood cells (WBC) were extracted from medical record system.

**Results:**

Serum PCT level was predominantly higher in gouty arthritis than in RA and AS patients, especially in the GA patients with tophi. PCT was significantly positively correlated with VAS, CRP and ESR in gouty arthritis and CRP in AS. PCT also had positive correlation-ship with ESR, DAS28 and ASDAS in RA and AS patients respectively, but significant differences were not observed.

**Conclusions:**

These data suggested that PCT is not solely a biomarker for infection, but also an indicator in inflammatory arthritis, especially in gouty arthritis.

## Introduction

Procalcitonin (PCT) is 116 amino acid polypeptide precursors to the calcium regulating hormone calcitonin and its synthesis is regulated by C cell of the thyroid gland, Calc-1 gene located on chromosome-11. Indirectly, PCT is also released from various form of pro-inflammatory cytokines such as IL-6, TNF- α, IL-1 etc and directly from microbial toxin during bacterial infections, thus systemic PCT often considered as acute phase biomarker of the inflammatory response [1,2]. It is detectable in serum and reaches to pick level within 25–30 hours after infection starts, decreases when infection is controlled and remains stable during normal condition (<0.05ng/ml) [3]. It is very important to differentiate infectious arthritis with aseptic arthritis such as gouty arthritis (GA), rheumatoid arthritis (RA) and ankylosing spondylitis (AS) in the early hours is often a challenging task for rheumatologist. GA is a repeated episode of inflammatory condition secondary to the high concentration of uric acid in blood associated with crystal deposition in connective tissues and presents as acute and chronic arthritis typically involvement of first metatarsal joint followed by other multiple joints, tophi, urolithiasis and renal disease [4]. It is one of the most common forms of arthritis in the US survey, the prevalence of gout among the US adults was 3.9% [5,6]. The prevalence of gouty arthritis in main land china in Han Chinese ranges from 0.15 to 0.67% and the trend of increasing incidence is more in southern cities of china [7]. Acute gouty arthritis is associated with high inflammatory clinical and biological symptoms, therefore prompt diagnosis and management is crucial [8]. Most of the studies have shown that diagnostic accuracy of serum inflammatory markers for infectious arthritis is significantly elevated level of serum PCT [3,9,10], serum CRP (C-reactive protein), ESR (erythrocyte sedimentation rate), synovial fluid WBC counts, and polymorphonuclear (PMN) percentage [11]. However, the role of PCT in non infectious gouty arthritis remains frontier. Interestingly, the diagnostic accuracy of PCT persists and unaffected by the uses of steroids, whilst the CRP is attenuated by steroid [12]. In a recent study has demonstrated that PCT levels in fresh synovial fluid are more sensitive and precise indicator compared to PCT level in fresh serum to distinguished between septic arthritis and non-infectious types of inflammatory arthritis [9]. In contrast, in our current study we found serum PCT level was predominantly higher in GA than in RA, AS patients and healthy control, especially in the attack of GA with tophi. All patients were active form of arthritis with median-high score of VAS, DAS28 and ASDAS without any evidence of infection in GA, RA, and AS patients respectively. Furthermore, PCT was significantly positively correlated with VAS, CRP and ESR in GA, but not with increased WBC level. As expected, PCT was not correlated with CRE (renal function), UA and WBC levels, which are often found abnormal in GA. Taken together, our data indicates that PCT is not only a marker of infectious state, but also a predictor for acute arthritis, especially in GA.

## Materials and Methods

### Ethics Statement

The study was approved by institutional research board (IRB) of the First Affiliated Hospital of Xiamen University. The written informed consents were obtained from all the participants: GA, RA, AS and Healthy volunteers. In case the participants had impaired ability to provide consent, written consents were obtained from the next-of-kin or the care giver on their behalf.

### Patients’ characteristics

All of the patients were Han Chinese selected from inpatient department of rheumatology and Clinical Immunology during years Feb, 2012 to Feb, 2015 in the First Affiliated Hospital of Xiamen University, Fujian, China. The GA subjects (n = 51, mean age 54.96 ± 12.03) including the attack of GA without tophi patients (n = 22, mean age 53.45 ± 15.04) and GA with tophi patients (n = 29, mean age 59.90 ± 11.06), RA (n = 37, mean age 55.35 ± 10.77), AS (n = 41, mean age 39.05 ± 17.14) and healthy control (HC) (n = 33, mean age 49.52 ± 12.03) were collected. The MSU tophi were confirmed by dual energy computed tomography (DECT) (Siemens Somatom Definition Flash). There was not a significant differences between in each age groups, except in AS and healthy control, it might be due to the prevalence of the early onset of disease in AS (Table 1). Moreover, necessary investigations were performed to rule out any evidence of systemic infection due to bacteria, fungus and virus in all patients. As we considered bacterial infection may coexist with urate crystals in the synovial fluid, if there is suspicious of infectious arthritis, synovial fluid tapped and cultured to rule out the infection (data not shown). We excluded the cases as follow, (1) With foci of infection any part of the body, (2) History of antibiotic taken before admission in the ward (3) Patient with various types of arthritis like osteoarthritis (OA), septic arthritis, Psoriatic arthritis, reactive arthritis etc were excluded. Hence, our inclusion criteria, the absence of infection had to be microbiologically confirmed or at least clinically not suspected.

**Table 1:** Characteristics of the GA, RA, AS and, HC groups.

Characteristics	GA		RA	AS	HC
**Total (Male/Female)**	51 (49/2)		37 (8/29)	41 (33/13)	33 (21/12)
**Age at study, mean (S.D.), years**	54.96 (12.03)		55.35 (10.77)	39.05 (17.14)*	49.52 (12.03)
	GA (none tophi)	GA (tophi)	NA	NA	NA
	53.45 (15.04)	59.90 (11.06)	NA	NA	NA
**Duration of disease, mean (S.D.), years**	9.58 (7.97)		10.03 (8.67)	6.41 (7.93)	NA
	GA (none tophi)	GA (tophi)	NA	NA	NA
	5.43 (4.97) **	12.72 (8.43)	NA	NA	NA
**VAS mean (S.D.)**	4.07 (1.15)		NA	NA	NA
**DAS28 (CRP) mean (S.D.)**	NA		4.97 (1.12)	NA	NA
**ASDAS(CRP) mean (S.D.)**	NA		NA	2.97 (0.81)	NA

* indicates AS vs HC *p* = 0.012,

** indicates GA (none tophi) vs GA (tophi) *p* = 0.0003.

GA: Gouty arthritis, RA: rheumatoid arthritis, AS: Ankylosing Spondylitis, HC: healthy control, VAS: Visual analogue scales, DAS28: disease activity and 28 joint disease activities score, ASDAS: ankylosing spnodolylitis disease activity score. NA: not analyze.

### Disease activity

Diagnostic criteria were completed each of the recruited patients accordingly GA (ACR 1997), RA (revised ACR 2010), AS (ACR1984). Visual analogue scales (VAS) 0–10cm were applied in GA (mean 4.07 ± 1.15) [13]. In RA composite disease activity measures included the clinical disease activity and 28 joint disease activities score (DAS28) mean value is 4.97 ± 1.12 [14,15] and in AS disease activity are decided as per ankylosing spnodolylitis disease activity score (ASDAS) mean value 2.97 ± 0.81 [16]. These indices were determined within 24 hours of admission of the each patient.

### Sample collections

Under aseptic precaution total of 5ml venous blood samples was drawn from a vein at elbow pit from each patient on the first day of admission before any treatment instillation. Serum was isolated by centrifugation at 3000 rpm for 5 minutes and PCT was analyzed.

### Determination of serum PCT level

Serum PCT levels (normal range < 0.05 ng/ml) was measured within 24 hours after sample collection by using an electrochemiluminescence immunoassay (ECLIA) (Roche, Cobas6000) instrument according to the manufacturer’s instructions.

### Routine laboratory parameters

Complete blood count (CBC), CRP, ESR, Biochemistry (Uric Acid level), and Kidney function (CRE) test along with other routine test were performed as per hospital protocols to rule out the systemic infections and data extracted from medical record system.

### Statistics

Data are reported as the mean ± SD. The Spearman correlation coefficient was constructed for the determination of linear relationships between PCT and CRP, ESR, VAS, DAS28, CRE, ASDAS, UA and WBC counts in patients. Mann–Whitney U-test for unpaired samples was conducted. Results were analyzed using the the Prism software 5.0 program (GraphPad, La Jolla, CA, USA). *p* value <0.05 in Mann–Whitney U-test and the Spearman correlation coefficient was accepted as statistically significant.

## Results

### Serum PCT levels and its correlation with inflammatory arthritis

**Fig 1:**
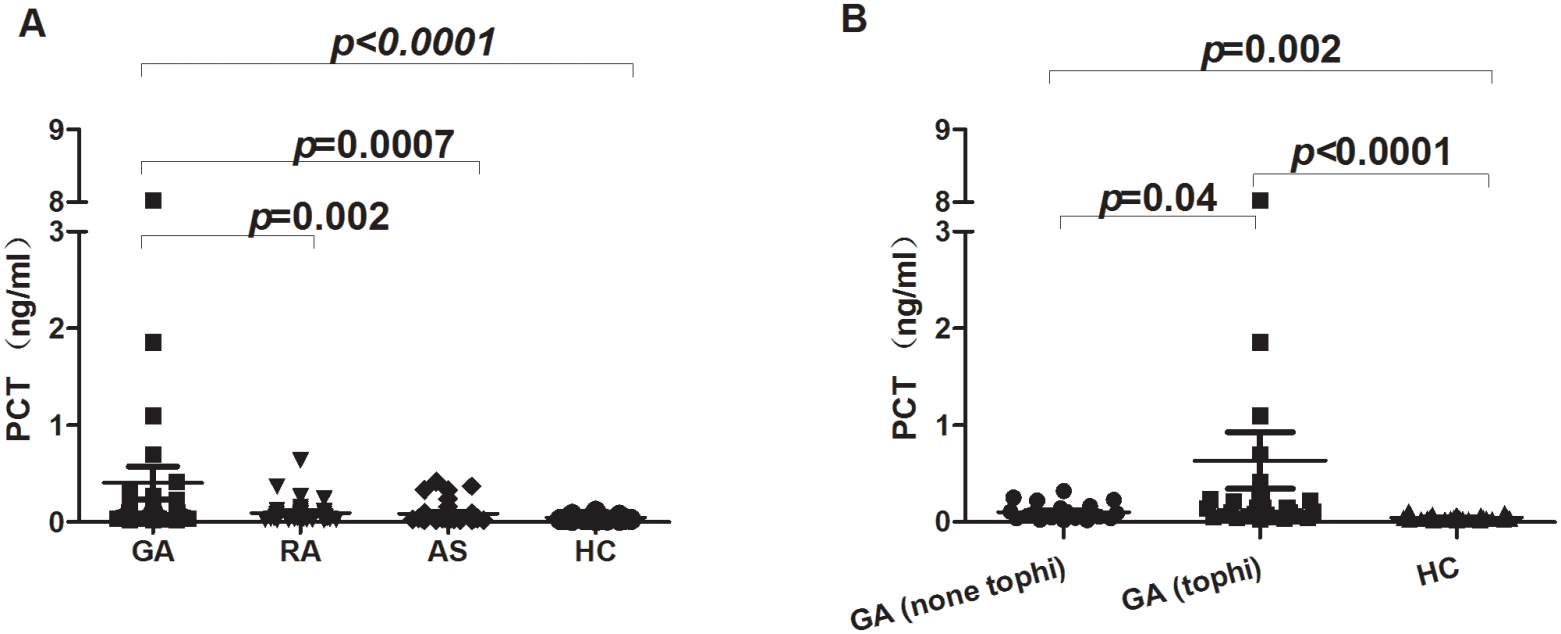
PCT is highly expressed in GA in comparison to RA, AS and Healthy control. (A) The comparison of serum PCT in GA (n = 51), RA (n = 37), AS (n = 41) and healthy control (n = 33) were determined by Mann–Whitney U-test. In GA patients PCT was significantly higher level observed than in RA (p = 0.002), AS (p = 0.0007) and healthy control (p<0.0001), whilst no significant differences in between RA and AS were observed. (B) The GA patients were divided into GA (tophi) (n = 29) and GA (none tophi) (n = 22) which indicates GA patients with tophi or not respectively. GA Serum PCT level was compared among these groups.

The serum levels of PCT in GA (0.41 ± 1.23, n = 51) is significantly higher when compared with RA (0.09 ± 0.12, n = 37) (*p* = 0.002), AS (0.09 ± 0.10, n = 41) (*p* = 0.0007) and HC (0.04 ± 0.02, n = 46) (*p*< 0.0001) (Fig 1A). MSU crystal deposits in multiple joints and tissue and form the tophi which act a critical role during the development of GA, thus the GA patients in this study were divided into GA with or without tophi. Expectedly, we found that a significant difference of PCT expression between the two groups (mean 0.64 ± 1.60, n = 29 Vs mean 0.10 ± 0.08, n = 22), *p* = 0.04 (Fig 1B). The serum PCT levels in RA (*p* = 0.06) and AS (*p* = 0.062) patients were not predominantly elevated when compared with healthy control, and there was also no significant difference in between RA and AS (*p* = 0.84) (Fig 1A). Taken together, these data showed PCT might not be the only marker for infection, but also an inflammatory indicator for aseptic arthritis, especially in GA.

### Serum PCT levels correlation with diseases activity indices, CRP, WBC and ESR of inflammatory diseases

**Fig 2:**
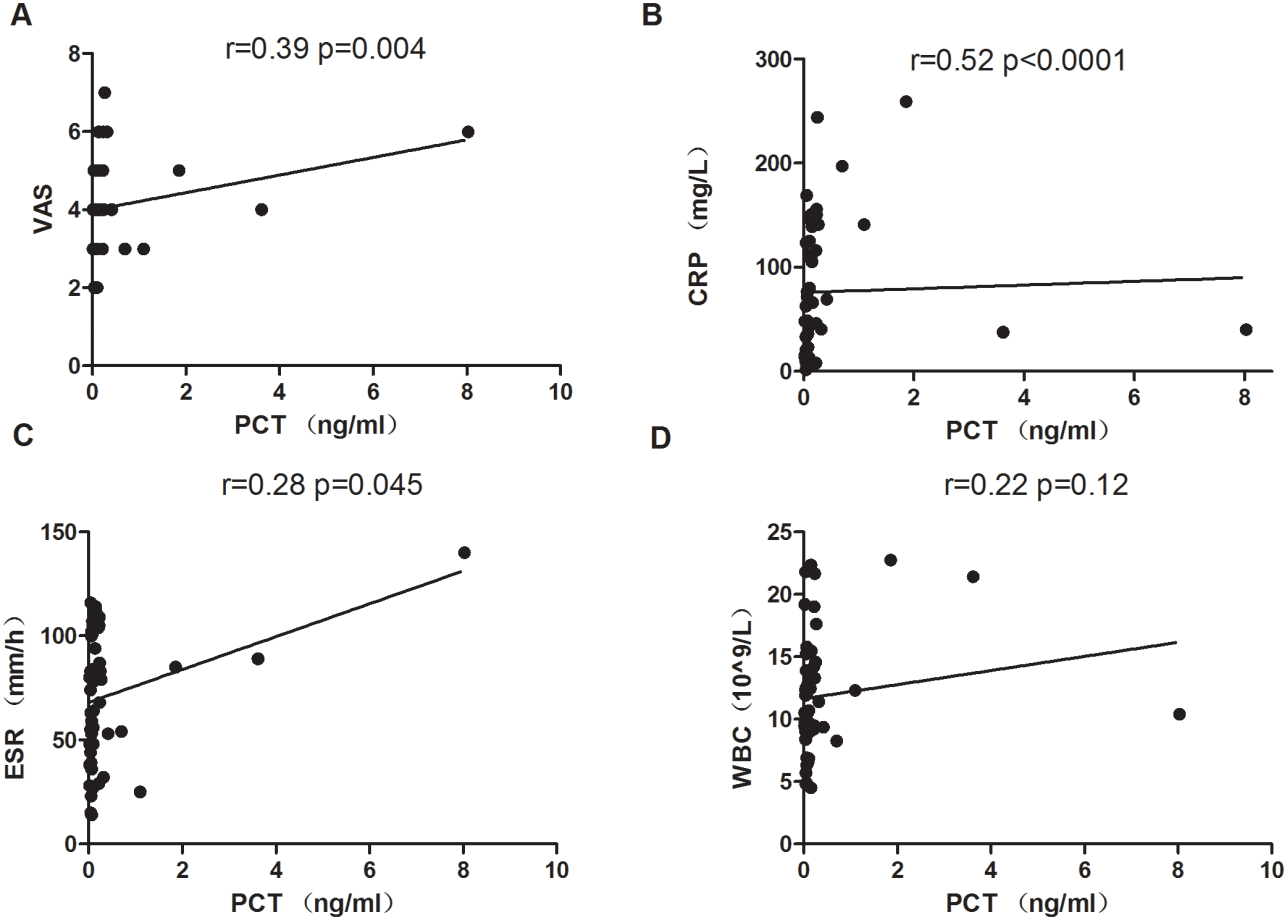
Positive correlation of serum PCT with VAS, CRP, ESR, and WBC in GA patients. The determination of linear relationships between PCT and CRP, ESR, VAS and WBC in GA patients (n = 51) was performed by Spearman correlation coefficient. PCT is significantly positively correlated with VAS (r = 0.39; *p* = 0.004), CRP (r = 0.52; *p*< 0.0001), ESR (r = 0.28; *p* = 0.045). There was no association between PCT and WBC.

**Fig 3:**
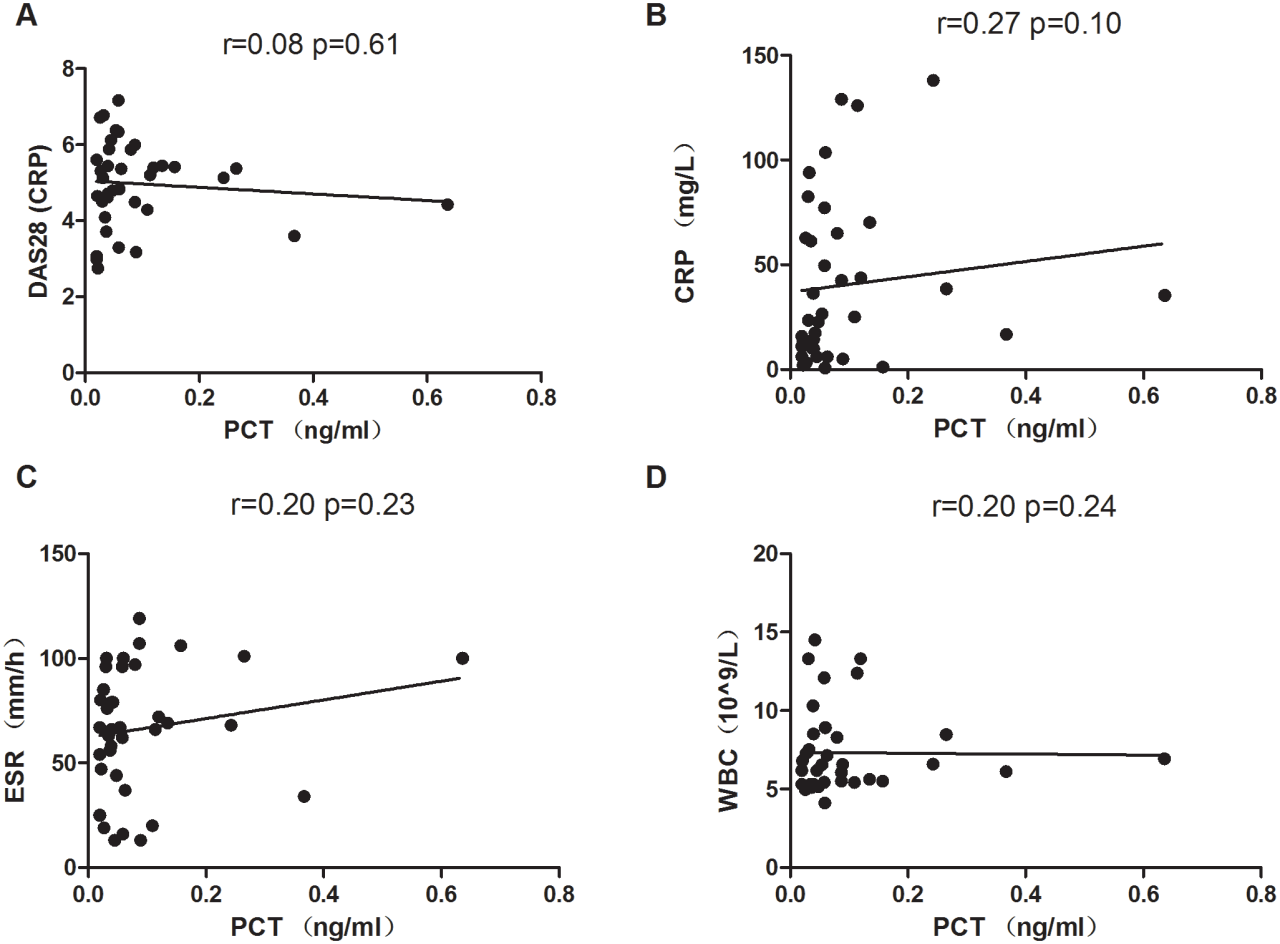
Correlation of PCT with DAS28, CRP, ESR and WBC in RA patients. The serum level of PCT was not significantly positively correlated with DAS28, CRP, ESR and WBC in RA patients (n = 37). Spearman correlation analysis was conducted.

**Fig 4:**
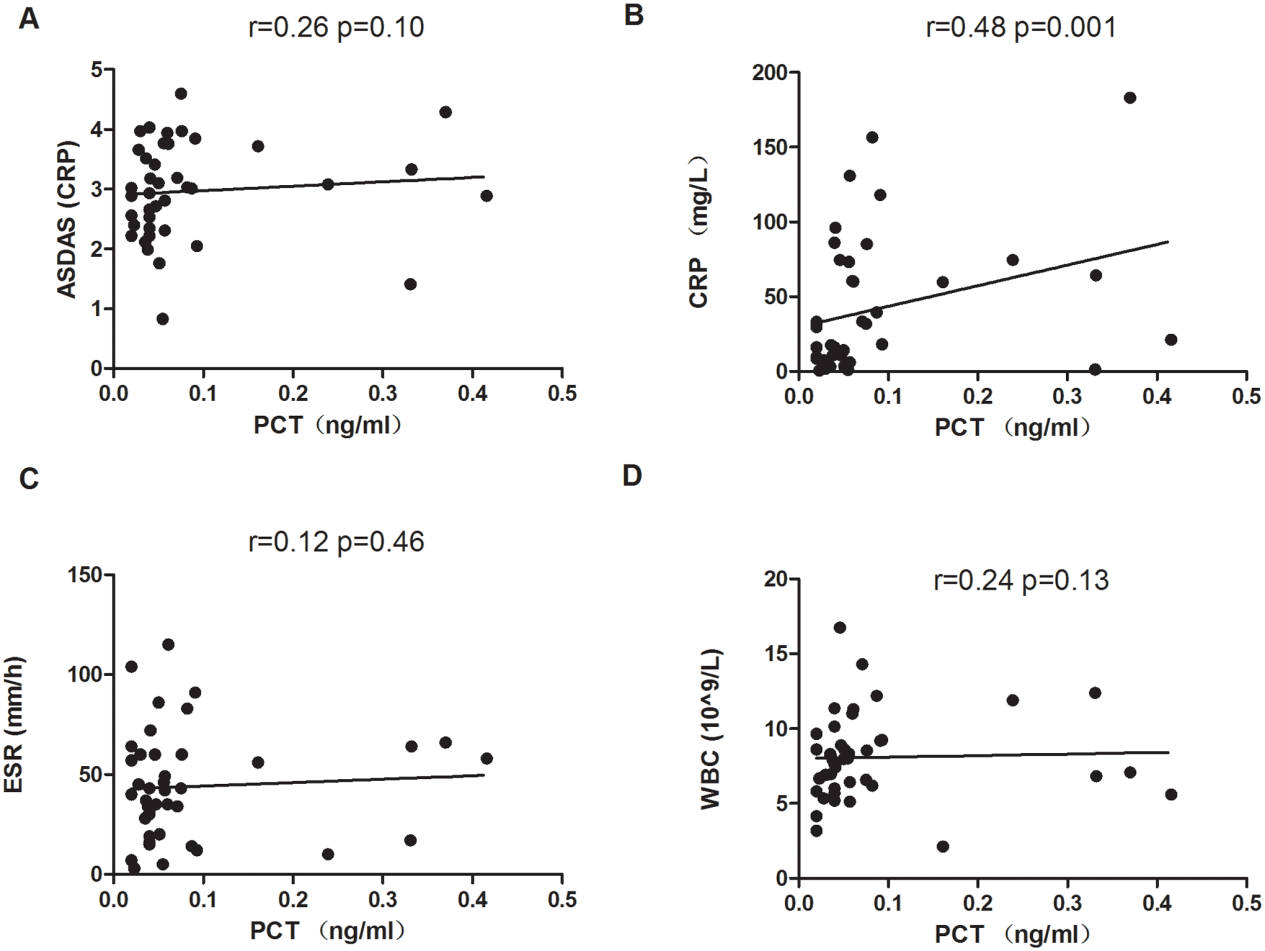
Correlation between PCT and ASDAS, CRP, ESR and WBC. Correlation of the serum levels of PCT with ASDAS, CRP, ESR and WBC was evaluated by Spearman correlation test in AS patients (n = 41). PCT was significantly positively correlated with CRP (P = 0.001). No correlation between PCT and ASDAS, ESR and WBC were detected.

Next, the correlation of PCT and inflammatory markers of arthritis were analyzed. In GA patients, PCT was significantly positively correlated with VAS (r = 0.39; *p* = 0.004) and CRP (r = 0.52; *p*< 0.0001). PCT was also significantly correlated with ESR (r = 0.28; *p* = 0.045) (Fig 2). The levels of PCT in RA and AS patients were positively correlated with disease activity indices DAS28 (r = 0.08; *p* = 0.61) and ASDAS (r = 0.26; *p* = 0.10), CRP (r = 0.27; *p* = 0.10/ r = 0.48; *p* = 0.001) respectively, no significant correlation was observed in RA and AS patients (Figs 3–4), except CRP in AS (Fig 4B). Furthermore, PCT levels in RA and AS were not significantly positively correlated with ESR (r = 0.20; *p* = 0.23/r = 0.12; *p* = 0.46). WBC is often elevated in the patients with infectious conditions, while no markedly positive correlation of PCT with WBC was found in our GA (r = 0.22; *p* = 0.12), RA (r = 0.26; *p* = 0.12) and AS (r = 0.21; *p* = 0.16). Besides CRP and ESR, our data suggested that PCT may be also a biomarker for an acute inflammatory arthritis.

### Serum PCT levels correlation with serum creatinine (CRE), uric acid and WBC

**Fig 5:**
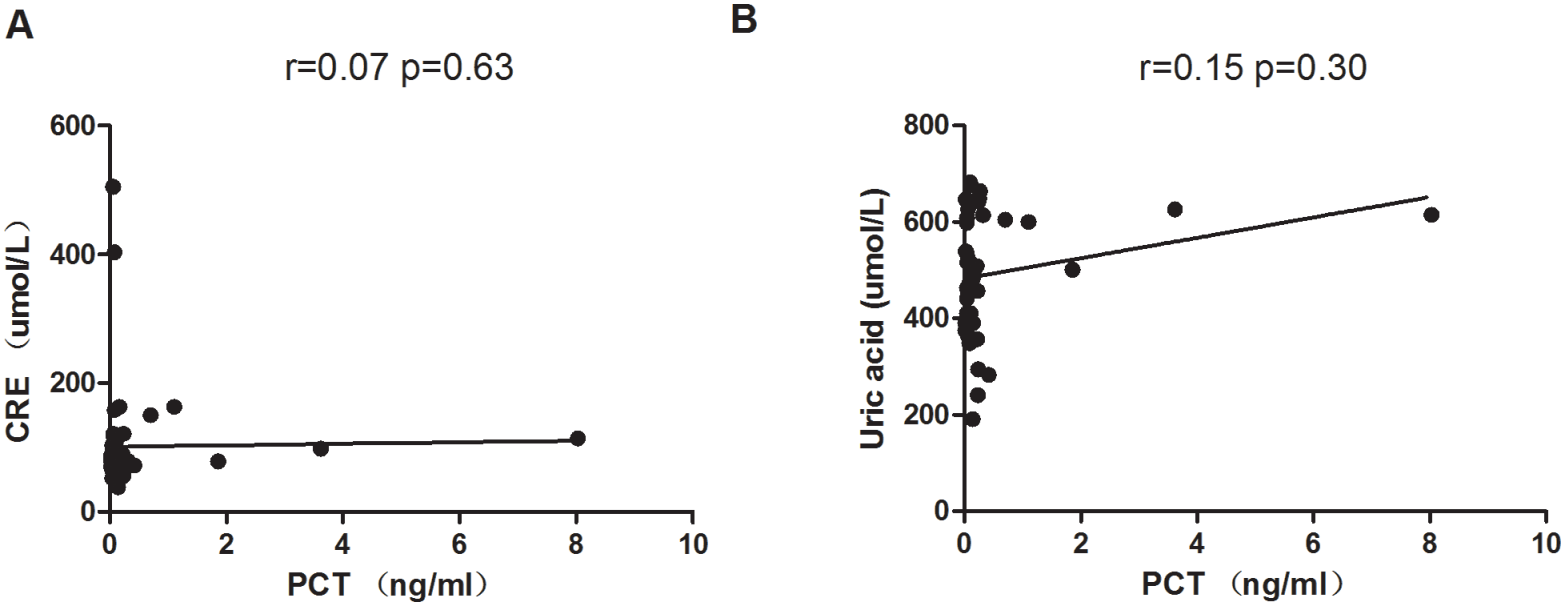
Correlation of PCT with CRE and Uric acid. Spearman correlation test was performed to analyze the correlation between serum PCT and CRE, uric acid levels in GA patients (n = 51). PCT was not correlated neither with CRE (r = 0.07, *p* = 0.63) nor Uric acid (r = 0.15, *p* = 0.30).

The renal functions often impaired when patient is suffering from GA, and biochemical markers such as CRE, BUN and UA often elevated in renal impaired patient. However, we found serum PCT level was not correlated with CRE (r = 0.07; *p* = 0.63) and uric acid (r = 0.15; *p* = 0.30). These data suggested that the increased levels of PCT are not resulted from the renal impairment in GA patients in our study (Fig 5).

## Discussion

The Differentiation of gouty arthritis from infectious inflammation is often a challenging job for rheumatologist in their daily practice. Most of the studies have been shown significantly elevated level of serum PCT as the diagnostic accuracy of serum inflammatory markers for septic arthritis [10,17,18]. The accuracy of the differential diagnosis of septic arthritis from GA and RA using serum PCT is significantly lower compared with that by synovial fluid PCT levels [9]. However, data concerning serum PCT levels on patients with active underlying systemic autoimmune diseases, inflammatory arthritis is limited. In the early hour to distinguish of infectious arthritis from non-infectious gouty arthritis is very important and sometimes only high PCT value is not useful indicative marker for their differentiation. Intriguingly, different inflammatory rheumatic diseases have afforded data regarding the usefulness of PCT. Elevated PCT levels in the absence of bacterial infections have been seen in patients with certain inflammatory conditions such as Kawasaki disease [19], Adult-onset Still’s disease (AOSD) [20] and some cancers like medullary carcinoma of the thyroid and small-cell lung carcinoma[1]. Consistently, in our study we have found that higher range of PCT level in GA compared to other inflammatory arthritis like RA (*p* = 0.002), AS (*p* = 0.0007) and healthy controls (*p*< 0.0001). We further compared the PCT levels in between acute attack of GA without tophi and with tophi, and found PCT level was significantly elevated in GA patients with tophi (*p* = 0.04) and both groups were considerably differences when compared with HC (Fig 1B). Previous studies have been revealed that concomitant septic and crystal induced gouty arthritis can occur and represented the acute attack of gouty arthritis. The coexistence of bacterial infection and urate crystals in the synovial fluid may be the mechanism responsible for complicating crystal arthropathy [21–23]. Nevertheless, considering these evidences if there was suspicious of infectious arthritis, synovial fluid tapped and cultured, but there was no any evidence of septic arthritis in our patients. A Previous studies have been shown that PCTs released from various form of pro-inflammatory cells, cytokines IL-6, TNF- α, IL-1, IL-2 etc, however pathways remains unclear [1,24,25]. Additionally, the deposition of monosodium urate (MSU) crystals within joints induces strong proinflamatory stimuli that can initiate, amplify, and maintain an intense inflammatory response through the release of proinflammatory mediators such as IL-1β, TNFα, and IL-8 [26,27]. These could be reason for raised PCT level in our gouty arthritis patients. Gout is a common arthritis caused by deposition of monosodium urate crystal, which could be one of the reason to clarify attack of GA patients with tophi have significantly higher levels of PCT than acute attack of GA without tophi. A study has revealed that in gouty arthritis, crystal-induced inflammation is interleukin-1 production by activation of the inflammasome [28], blocking of IL-1 showed rapid decreases of VAS and CRP levels [29]. However, we have not compared serum TNF- α, IL-6 and IL-1 levels in our study groups, GA is often associated with significant elevation of CRP and ESR during acute attack [29,30]. In our study patients serum PCT level was significantly positively correlated with VAS (r = 0.39; p = 0.004), CRP (r = 0.52; p< 0.0001) and ESR (r = 0.28; p = 0.045) in gouty arthritis. Soderquist B et.al has identified that serum CRP levels are significantly higher during admission time in patients with cultured- verified infectious arthritis and polarizing microscopic verified crystal-associated arthritis but peripheral WBC counts and PCT levels are not differ in between two groups [31]. In contrast PCT values were more discriminative than WBC and CRP in distinguishing a bacterial infection from another inflammatory process though it has low sensitivity [32,33]. In our study, the serum PCT level was not notably increased even in moderately active form of RA (DAS28 = 4.97 ± 1.12). Some studies also revealed that low levels of serum PCT were presented in low grade of inflammation in seropositive arthrialgia, non infectious RA patients [9,34]. Consistently, our study showed PCT was not positively correlated with CRP, ESR and WBC in RA (Fig 3). Therefore, high PCT level is more specific marker than CRP, ESR and WBC counts for the detection of bacterial infection in RA patients [35]. In AS patients and controls serum PCT levels are not significantly higher and these values are independent with diseases activity and medications [36]. Similarly, serum PCT level was also not significantly increased in our AS patients, whilst PCT was positively correlated with CRP. PCT and degree of renal impairment has shown no correlation in systemic autoimmune diseases without systemic infection [37]. Here, in our study we also showed that there is no significant correlation between serum PCT and CRE levels. Previous studies have shown that biochemical markers such as CRE, BUN (blood urea nitrogen) and UA are often elevated and directly linked to the impairment of renal function in GA patients. However, we found serum PCT level was not correlated with CRE and UA. We assumed that PCT levels were not elevated due to renal dysfunction. Importantly, a study has shown that renal elimination of procalcitonin is not a major mechanism for procalcitonin removal from the plasma[38]. In most of the autoimmune diseases without systemic infection, the serum PCT levels are within normal range (<0.05ng/ml) but CRP can be elevated in patients with active underline disease. However, in our study we found that increased PCT expression in the acute attack of GA that might be due to inflammatory cytokines induced by MSU release, while moderate active form of RA and AS patients PCT was not significantly elevated.

Limitations of this study are the small number of patients of inflammatory arthritis. Nonetheless, all recruited cases in our study were free of infections. We did not analyze crystal pattern of GA patients, considered gout is a common arthritis caused by deposition of monosodium urate (MSU). In the absence of a bacterial infection elevated PCT levels were found in condition with severe stress for example, major trauma, operations, cardiac shock etc [39]. However, these conditions are very unlikely to be come across in routine rheumatology practice and none of the above evidence found in our cases.

In conclusion, our study suggested that the high serum PCT levels, like CRP is also associated with inflammatory gouty arthritis without infection susceptibility in southern Chinese Han population. However, further study should be done in a larger sample size and other ethic to test and verify our result.
